# Advancements in Biomedical Applications of Calcium Phosphate Glass and Glass-Based Devices—A Review

**DOI:** 10.3390/jfb15030079

**Published:** 2024-03-21

**Authors:** Jawad T. Pandayil, Nadia G. Boetti, Davide Janner

**Affiliations:** 1 Department of Applied Science and Technology (DISAT), Politecnico di Torino, Corso Duca degli Abruzzi 24, 10129 Torino, Italy; jawad.talekkara@polito.it; 2 LINKS Foundation-Leading Innovation and Knowledge for Society, via P. C. Boggio 61, 10138 Torino, Italy; nadia.boetti@linksfoundation.com

**Keywords:** bioresorbable, calcium phosphate glass, polymer composites, ion release, therapeutic agents, drug delivery, reinforcing agents, resorbable optical fibers, biomaterials, optogenetics

## Abstract

Calcium phosphate (CaP) glass has recently gained popularity as a promising material for a wide range of biomedical applications. Recent developments have seen CaP glasses moving from a passive implant material to an active degradable material, particularly as a major constituent of bioresorbable photonic devices. This holds great promise in advanced biomedical applications, since the main constituents of CaP glasses are present in the human body. In this review, the progressive advancements in the biomedical applications of calcium phosphate glass-based devices over the past 50 years are discussed. An overview of their role as reinforcing agents and the studies on doping their matrices for ion releasing and drug and gene delivery are reviewed. Recent applications of CaP glass and fibers in soft-tissue engineering and their potential for optical quality bioresorbable devices are then discussed along with the current challenges and potential future directions, emphasizing the promising role of CaP glass in the next generation of biomaterials. Considering their progress and potential in performing several biomedical functionalities over time, CaP glass-based devices hold promise for becoming enabling tools as an implantable, bioresorbable, multifunctional class of devices in future biomedicine.

## 1. Introduction

The field of biomaterials encompasses a wide range of materials classes, including metals, ceramics, polymers, glasses, and composites. Those have been studied over time to repair and replace hard tissues or improve tissue functions in the human body. Even before the development of the science of biomaterials, practices of bone fracture fixation existed, and evidence of them was discovered in the early 1900s during the Hearst Egyptian expedition of the University of California [1,2]. Indeed, they found two fractured bone specimens; one was an adolescent femur with a mid-shaft fracture, and another specimen was an open fracture of a forearm. The first specimen was splinted with four wooden boards wrapped in linen bandages to fix the fracture, and the latter was found to be treated by the vegetable fibers of date palm adhered to the upper ulna fragment, evidently having been pushed to the wound to stop the bleeding. Similarly, the techniques of autografts and xenografts used to repair damaged tissues also possess a more extended history, as they date back to ancient times. Autograft refers to a graft of tissue from one point to another of the same individual’s body, e.g., the transplantation of a piece of bone. At the same time, xenograft is a transplant from the donor of a different species, mainly from porcine and bovine sources. Despite the successes of bone graft transplantation by the 1920s, complications of bone grafting are relevant, mainly due to the limited material availability for the case of autografts [3]. The xenografts (or allografts) integrate into the body more slowly than the autografts. They can also possibly bring the risks of infection, blood clotting, and other possible contaminations and antigenic problems, as bone composition and properties vary considerably within a population and are difficult to control [4].

Synthetic bone-grafting products were then developed from the 1960s, such as metals, ceramics, polymers, and composites, offering controlled composition and properties that can be adapted and optimized for bone replacement [5,6,7]. They are relatively easy to store, shape, and sterilize, and there is no need for the complex organization of bone organ banks. These materials can be referred to as the first generation of biomaterials, where the main goal was to attain the physical properties matching those of the replaced tissue with minimal cytotoxic effects [8]. They mainly included Pt, Pt-Ir wire electrodes, and PMMA for sensory and neural systems, as well as stainless steel as bone plates, screws, and wires. Silicone gel and rubbers were used for space-filling soft-tissue prostheses [9,10]. These materials were mainly ‘biologically inert’ and were already in clinical use by the 1980s in the United States.

During the late 1960s, there was a high interest in the production of ‘bioactive’ components that could elicit a controlled action or reaction in the physiological environment or resorb in the body over time, apart from being exclusively passive or inert [8]. These components, also referred to as the ‘second generation of biomaterials’, positively interact with the biological environment. Few of the polymers and glasses even resorbed in the body, where the implant was gradually replaced by regenerating tissues, mitigating, to an extent, the existing tissue–implant interface issues of first-generation biomaterials, which arose from the significant mismatches in mechanical moduli [11,12]. This was achieved by improving the material and bone interface by introducing either mechanical or chemical bonds. Unlike the biomaterials in the first generation, the second generation of biomaterials possessed porous and textured surfaces, where the ingrowth of bone or tissues into the pores helped in permanent fixation. As the pore fraction of the implant can affect its mechanical strength to a significant extent, methods that chemically bond between the material and the bone were then introduced, where the hydroxy apatite interfacial bonding offered a potential solution through direct chemical bonding [13].

Porous and textured surface calcium phosphate glasses were studied during this time to assess their biocompatibility and bioactivity, especially their interactions with bone tissues [13,14]. They were found to be resorbable, biocompatible, and osteoconductive, supporting the proliferation and differentiation of cells. By altering the composition of the phosphate glass, it was possible to tailor the bioactive properties of the glass, making it an attractive candidate among second-generation biomaterials. At the beginning of the 1980s, the clinical use of various bioactive glasses and resorbable polymers such as PLA (polylactic acid) and PGA (polyglycolic acid) in fracture- and bone-fixation plates and screws became routine [9,15,16].

**Figure 1:**
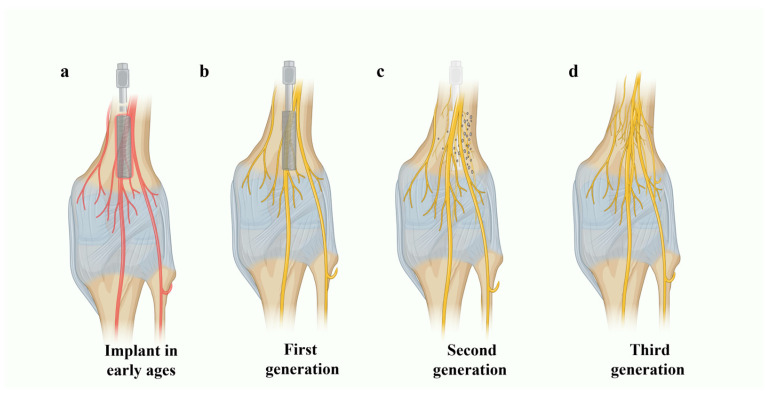
Schematic illustration of the various stages in biomaterials development explained using a screw implanted in the knee joint model. (**a**) Implant during the early ages with inflammation and cytotoxic effects (knee nerves appearing red). (**b**) First generation of passive implants with minimal cytotoxic effects. (**c**) Second generation of biomaterials, which are either bioactive (nerve or bone tissue proliferation) or bioresorbable. (**d**) Third generation of biomaterials, where the concepts of resorbability and bioactivity are combined (the implant was eventually dissolved and the space was filled by regenerated nerves/tissue) (created with BioRender.com).

By the 1990s, the molecular biology revolutions, along with the advances in genomics, promoted the development of the third generation of implantable devices. The concepts of resorbability and bioactivity were combined into the same material to form bioactive glasses or composites [17]. The transition from the second generation to the third generation was influenced mainly by the choice of material design to activate genes for tissue regeneration, along with the proper choice of the composition that alters the solubility rate and the percentage of available active ions [9,13]. These “intelligent” materials are bioinstructive, which elicit specific interaction with the cell surfaces, inducing cell proliferation and helping the body heal itself once implanted [9]. Various stages in the development of biomaterials are schematically depicted in Figure 1. Several physical forms of CaP glass, such as disks, bone plates, rods, screws, nails, fibers, and microspheres, were developed and studied during these times, where the possibilities of doping the glass systems with therapeutic ions or proteins to promote the bioactivity were investigated later in several studies [18,19,20,21,22,23].

After the 2000s, with significant advances in material science, manufacturing, bio-, and nanotechnology, along with the enhanced understanding of electrophysiological behavior at the cellular level, research towards the development of a new generation of miniaturized devices capable of performing biological, electrical, and photonic functionalities within the single device gained popularity [24]. Development of a fourth generation of biomaterials which are multifunctional and can simultaneously stimulate, manipulate, and record bioelectrical and optical activity in vivo will have significant implications for the concept of personalized and predictive medicine, also taking into account the improvements in data analytics [25,26]. Among the different classes of materials mentioned, glass–ceramics offer the best potential in altering the chemical composition or varying the percentage and size distribution of porosity [13]. Novel methods of their room temperature-based preparation methods also bring flexibility in terms of the type of drug or molecules that can be incorporated in the glass matrix to make them multifunctional [27]. In this context, the focus of this review is on the bioactive glass materials, especially calcium phosphate.

While significant work has been performed over the years to elucidate the role of phosphate glass in different biomedical applications, there is a lack of data correlating these advancements. This review focuses on the eventual advancements in applications of CaP glass in biomedicine, starting from its role in the second and third generations of biomaterials. We will then discuss the multifunctional third generation of CaP glass-based implants and the recent advances in CaP glass-based photonic devices, making them potential candidates for the development of the fourth generation of biomaterials.

## 2. Methodology

**Figure 2:**
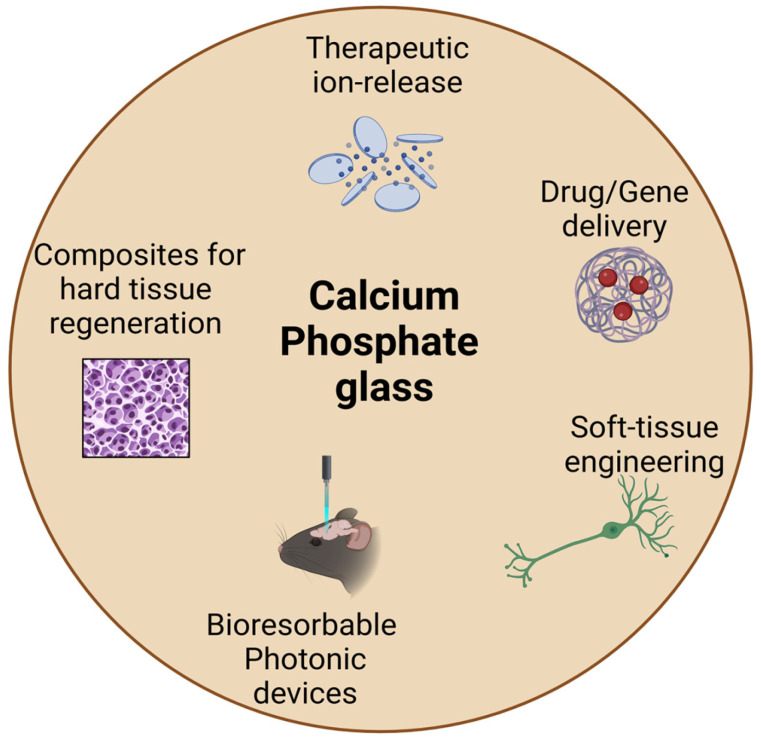
Biomedical applications of CaP glass discussed in this review (created with BioRender.com).

PubMed and Scopus were the electronic data bases used for this review. Scientific articles related to the biomedical applications of calcium phosphate glasses published between the years 1971 and 2023 were manually selected based on their abstract analysis. Articles conveying similar ideas were excluded and the selected articles were then divided into the categories shown in Figure 2 based on content analysis. These categories were then assigned under the titles of the second, third, and fourth generations of biomaterials based on the specific qualifying criteria mentioned in the introductory section for each generation.

## 3. CaP Glass in Second- and Third-Generation Biomaterials

Calcium phosphate glasses having different Ca/P molar ratios (5, 1.8, and 0.5) in their compositions were developed and studied in the 1970s by L.L. Hench et al. [13]. The in vivo study on the rat femur showed that the glass implants were intimately united to the bone after 6 weeks from implantation without any inflammatory response in the animal tissues. The in vitro studies assessed the behavior of the glass in parallel in an acidic environment, and the glass was found to be dissolving. Considering this is among the earliest studies in the development of CaP-containing glass and was reported to provide a better bonding interface to the bone and was soluble in the physiological medium, they already qualify the criteria to be considered a second-generation biomaterial.

Even though there were a variety of metals and bioresorbable polymers finding clinical use during these times, they suffered from Young’s modulus and the tensile strength being considerably higher or lower than that of the cortical bone, causing wear stress, as can be seen in Table 1. The bone does not carry sufficient load during the healing process due to the rigid metallic implant taking the load, which can cause the bone to fracture again during the extraction of the metallic implant. Metallic implants can also have corrosion products, which may adversely affect the local tissue reactions [27]. The implants prepared from biodegradable or bioresorbable polymers and glasses, engineered to degrade at a rate that would slowly release the load into the healing bone, could be a solution. Regarding the glass implants, several studies were reported between 1970 and 1990 on analyzing the long-term stability and biocompatibility of CaP glass–ceramic implants, their interface with the bone, and the compositional dependance of bioactivity and cytotoxicity [28,29,30,31,32,33,34,35,36], and a few of them are detailed in Table 2. Concerning the polymers, many of them, such as PLA and PLGA (polylactic-co-glycolic acid), possess relatively lower mechanical properties to be used in hard tissue repair applications for load-bearing purposes [37,38].

### 3.1. CaP Glass as Reinforcing Agents

Reinforcement was required to produce an implant with mechanical properties that almost matched those of the bone tissues. The suitability of calcium phosphate glass-based optical fibers to be used as reinforcing agents was the subject of research from the late 1980s, and the idea was patented in 1986 [30]. The fiber-reinforced composites, such as poly (glycolic acid) and calcium–aluminum phosphate glass fibers, possessed good strength but poor hydrostability [14]. The effort to increase the fiber–polymer composite’s durability succeeded in the trials incorporating Fe_2_O_3_ in the fiber composition. ST Lin et al. found that the iron oxide content strongly affected the dissolution rate of the glass, as well as the cell attachment and differentiation. These durable-strength fibers were found to be important as a reinforcing agent for developing bioabsorbable composite implants.

#### 3.1.1. CaP Glass-Based PCL Reinforcement

PCL is a polyester that has been widely used in the tissue engineering field for its availability, relatively inexpensive price, and suitability for modification. They have collected much attention among other biodegradable polymers to produce films, mats, drug-delivery systems, and scaffolds for various tissue engineering applications. Many studies have proven that the incorporation of ceramic particles into the PCL matrix enhanced their mechanical strength while introducing osteoconductivity and bioactivity [41].

The effect of phosphate glass reinforcement on PCL degradation was systematically investigated by Alani et al. in [42]. Compared to the PCL-alone homopolymer, whose weight remained generally constant, the PCL-PG composite underwent a weight loss of around 5–20% depending on the glass composition. The composite containing 24 mol% of Ca in the phosphate glass composition lost 20% of its weight, while the 36 mol% composite lost approximately 4% of its weight over a duration of 600 h. A reduction in stiffness was also observed for the composite in proportion to the weight loss. The study confirmed the possibility of producing a biomaterial whose stiffness and weight behavior may be controlled, and possibly even manipulated, bringing immense potential for the PCL-PG composites in tissue engineering applications. The phosphate glass particles used in this study (45 mol% P_2_O_5_-based) were prepared by agate milling the casted glass for 1 h and then ball milling for 24 h to ensure the glass breakdown into small and even fragments. Finally, the glass particles were sieved through a 150 mm particle size sieve, and solvent dispersion produced the composite [33]. The PCL-PG composite prepared in the same way has been investigated as a filling material for root canals in a later study [43]. Within an aqueous environment, the composite releases specific ions (Na^+^, Ca^2+^, [PO_4_]^3−^, [P_2_O_7_]^4−^, [P_3_O_9_]^3−^, and [P_5_O_10_]^5−^) at controlled rates. These composites adhere better to the canal wall than the conventional Gutta-percha (GP) filler due to ion precipitation on the canal wall, forming a continuous film with continuity between the composite and canal wall. This study demonstrated the potential of a PCL–phosphate glass composite as a root-filling material capable of producing a seal in an aqueous environment without an additional sealer.

**Figure 3:**
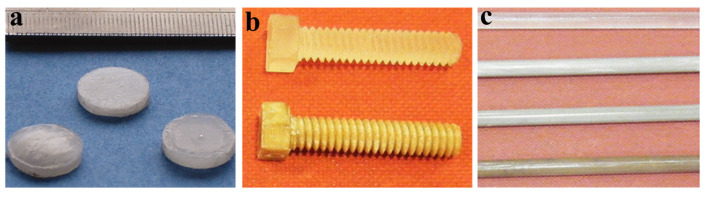
(**a**) Polycaprolactone (PCL)–phosphate glass disks before implantation (8 mm in diameter) (reprinted with permission from [44]). (**b**) Screw from PLA (polylactic acid) alone (top), and screw of PLA–phosphate glass fiber composite (down) (reprinted with permission from [45]). (**c**) PLA-PGF composite rods (top: PLA alone and down: PLA-PGF in the order of P50RM, P50UD, and P40UD rods, where RM stands for random fiber mats and UD stands for unidirectional fiber mats, while P40 and P50 are 40 and 50 mol% of phosphate in the composition) (reprinted with permission from [39]).

PCL-PG composite disks of 8 mm in diameter and 1 mm in thickness were implanted in rats to test the repair of calvarial defects in a later study by A. Scotchford et al. (Figure 3a) [44]. Similar to the degradation behavior observed in the previously mentioned study of Alani et al., the composites showed more severe evidence of degradation, with small-crevice formation and irregular implant edges, providing an opportunity for the tongues of tissue ingrowth compared to the PCL-alone disks. The fiber-reinforced disks demonstrated a 15% increase in mineralized bone over time compared to the PCL-alone disks. No inflammatory responses or clinical complications were observed, showing the biocompatibility of the composite, and the histological examination revealed extensive bone growth after 25 weeks of implantation. The study has allowed them to confirm that the composite material showed considerable promise for progression to trials in patients. The phosphate fibers (15 µm in diameter) used in this study were produced in-house using a melt-drawing system.

Studies to analyze the effect of calcium phosphate reinforcement on the PCL polymer’s surface morphology were later carried out with a nanocrystalline calcium phosphate (CP) ceramic coating. The CP coating provided randomly oriented thorn-like particle structures of a nm size, forming a very porous structure similar to that in the natural bone [46]. They also prepared biomineral-doped CP (dCP) by adding Mg (1.61 mg/L), Zn (0.46 mg/L), and Sr (0.28 mg/L) in distilled water during the preparation of the CP. The Ca/Mg/Zn/Sr ratio of 97:2.5:0.45:0.05 in weight percent was set to mimic the range of the trace element concentration in bones. This dCP was used to prepare CaP-PCL composite coatings with increased roughness in the implant material, which was found to be very favorable for bone cell adhesion, with a denser structure and the maintenance of a porous nature.

#### 3.1.2. CaP Glass-Based PLA Reinforcement

PLA, also known as polylactide, is a versatile biopolymer consisting of lactic acid monomers. Since its first recorded synthesis in 1932, followed by subsequent advancements, it has shown promise as a biomaterial in a plethora of healthcare applications, such as tissue engineering or regenerative medicine, cardiovascular implants, dental niches, drug carriers, orthopedic interventions, cancer therapy, and skin and tendon healing. PLA has been approved by the FDA (Food and Drug Administration) for direct contact with biological fluid. Semi-crystalline PLA exhibits an approximate tensile strength of 50–70 MP and a flexural modulus of 5 GPa, and researchers continue to explore the possible modifications to match the properties for specific biomedical applications [47].

PLA has been reinforced with PGFs in several studies to investigate the improvements in mechanical properties, as can be seen in Table 3. In [48], Delia S. Brauer et al. embedded phosphate fibers into a degradable organic polymer network based on methacrylate-modified oligolactide (a component of PLA) and conducted some preliminary tests to use it as a fracture-fixation device. The three-point bending test of the composite showed an elastic modulus of 16 GPa, which is in the range of the elastic moduli of cortical bone. The study envisaged to improve the modulus value in future studies using a higher volume fraction of the fiber with smaller diameters, which might be desirable to prevent bone motion during the healing process. However, the SEM image revealed broken outer fibers in the composite, delamination, branching cracks, and fiber pull-outs during the mechanical tests. This indicates the need for improvements in the interface between the polymer matrix and glass fillers, which could be possible by chemical bonding or the physical interlocking between the matrix and fibers. A better interface could also prevent the rapid deterioration of mechanical properties in the cases of the reinforcement with completely degradable fiber.

In the biocompatibility studies using murine MC3T3-E1 pre-osteoblast cells, the cells adhered on the composite surface, proliferated, and grew into a continuous cell layer. Hence, the material served as a suitable support for the pre-osteoblast cells. Fibers (35 P_2_O_5_–27.5 CaO–9.5 MgO–22.5 Na_2_O–5.5 TiO_2_) in this study were produced using a preform technique. The fibers (125µm) were drawn from the rod-shaped preform.

**Figure 4:**
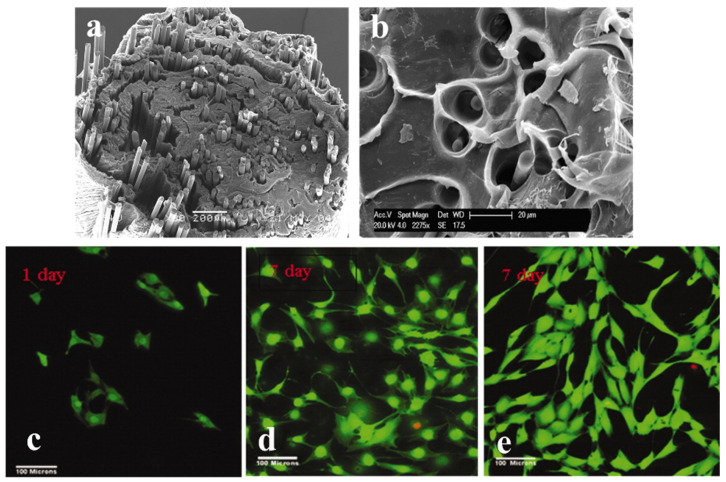
(**a**) Scanning Electron Microscope (SEM) image of the cross-section of PGF–collagen spiral constructs with the PGF generated at 25 Hz SMF (reprinted with permission from [51]). Scale bar: 200 µm. (**b**) Fracture surface sample of a heat-treated PLA/PBG fiber composite after 6 weeks of degradation in deionized water at 37 °C [49]. Scale bar: 20 µm. (**c**) Cytocompatibility assessment using MG63 cells on a PLA-PGF (heat-treated) composite surface on day 1, scale bar: 100 µm. (**d**) on PLA alone during day 7 (Scale bar: 100 µm.), and (**e**) on PGF (heat-treated)–fiber/PLA composites on day 7, stained with calcein AM to visualize the live cells green and with propidium iodide to see the dead cells red (reprinted with permission from [49]). Scale bar: 100 µm.

In [49], I. Ahmed et al. developed a fully degradable iron phosphate glass fiber–PLA composite (Figure 4b) and evaluated the mechanical and degradation properties along with the cytocompatibility studies. The composite contained a fiber volume fraction of 14%. Fiber reinforcement increased the flexural strength and elastic modulus properties compared to polymer alone. The strength profile of the composite produced in this study can be matched with the cortical bone, but the modulus values were lower than those required for the cortical bone, as mentioned in Table 3. Along with the composite degradation during the in vitro tests in deionized water, the mechanical properties were also significantly degraded (from 5 GPa to 1 GPa). The authors stated that these composites were produced without coupling or sizing agents, and a relatively weak interface might be anticipated, causing the disruption of the polymer–fiber interface during the degradation process. In vitro studies conducted on MG63 cells showed the good cell compatibility of the composite material (Figure 4d). Heat-treated fiber composites showed higher cell viability than non-treated fiber composites due to their slower degradation profile (14% mass loss for the non-treated and a 10% mass loss for the heat-treated fiber composites in 6 weeks). The fibers used in this study were produced by melt-draw spinning using a dedicated in-house facility traversed onto a Teflon sheet-coated metal drum.

In a later study, Felfel RM et al. investigated the use of novel phosphate glass fiber-reinforced rods as resorbable intramedullary nails [39]. The composites were prepared with different fiber architectures (random and unidirectional) with a 40 mol% P_2_O_5_ (P40) composition, where these fibers were molded into composite rods by forging at 100 °C.

The composite rods (Figure 3c) produced using long fibers parallel to the length of the rod displayed superior mechanical properties not only against the PLA alone but also against the cortical bone, where the flexural modulus for the unidirectional composite rods with a fiber mass fraction of 50 and 40% was 450 and 600% higher, respectively, in comparison to the pure PLA rods. Both the orientation of the polymer chains in the matrix and the fiber rearrangement during the rod manufacturing process were suggested to be responsible for improving the mechanical properties. They claim that these unidirectionally reinforced phosphate glass fiber–PLA composites have great potential to be used as intramedullary-fixation devices. The fibers in this study, having a diameter of 15 µm, were produced via a melt-draw spinning methodology using a dedicated in-house facility.

Na Han et al. used the same composition of the composite as in [48] to assess the effect of screw holes on them [52]. The flexural strength of the composite proportionally increased with the fiber volume fraction. The degradation mass-change studies showed that the 25 volume % of samples had the lowest and the 45-volume fraction % of the samples had the highest degradation rates within the first 10 days of immersion in PBS (phosphate-buffered saline solution). The effect of 2 mm screw holes did not drastically reduce the mechanical properties of the polymer–fiber composite, but the holes made them degrade faster. Bioresorbable screws (Figure 3b) were also developed from PLA reinforced with phosphate glass fibers, where the maximum flexural properties for the composite screw increased by 100% in comparison with the PLA screws [45]. Unidirectional fiber-reinforced composites produced superior mechanical properties than randomly oriented fiber composites.

#### 3.1.3. CaP Glass-Based PAA Reinforcement

PAA is a synthetic polymer made of an acrylic acid (AA) monomer. They feature pendant carboxylic groups, which are known for the remarkable cellular adhesion and proliferation features of PAA. PAA has been integrated with many polymers and sculpted into various architectures, namely, hydrogels, tablets, microspheres, nanoparticles, and microgels, to accomplish their biomedical potential [53].

Hybrid nanogels containing CaP nanoparticles and PAA were synthesized by an ultrasonically assisted precipitation method using a high-energy ultrasound probe for a controlled drug-delivery application in a study by Fang Li et al. [54]. These PAA/CaP hybrid nanogels showed good stability in biological media, with no evidence of hemolysis and cytotoxicity to L02 cells. They were loaded with the doxorubicin hydrochloride (DOX) drug, and the drug release was monitored under different PH conditions (4, 5, and 7.4), showing no abrupt release. The in vitro inhibition of cell proliferation using free DOX and DOX-loaded PAA/CaP hybrid nanogels was assessed in HepG-2 cancer cells, demonstrating that the anticancer effect of DOX was not lost after the incorporation in PAA/CaP hybrid nanogels. However, it was delayed due to the controlled release of DOX from the carriers. The passive tumor-targeting ability of the nanogel was confirmed by the in vivo tissue fluorescent imaging of mice with subcutaneously transplanted tumors.

In a later study, Qiang Chen et al. [55] demonstrated the innovative exploitation of the electrophoretic deposition method to develop textured surfaces by orienting the same fiber composition of [39] on stainless steel. This macroscale deposition of the sPGF-PAA (poly acrylic acid) composite coatings showed great potential in the directional functionalization of metallic implants. In addition, highly aligned sPGF in the composite coating enhanced osteoblastic proliferation and regulated cell migration and spreading in a one-dimensional orientation. Investigations have not reported on the effect of the oriented sPGF-PAA coatings on bone bonding in vivo.

#### 3.1.4. Phosphate Fiber–Collagen Composites

Collagen is a biological polymer which has been used as a biomaterial since 1881. Beyond its applications in wound healing, tissue engineering, and supplementation, demanding modification procedures to improve its physicochemical and mechanical properties for final application is still an area of ongoing research.

Phosphate glass fibers were used to create microchannels within plastic-compressed collagen gel, where PGF degradation was measured through ion chromatography, and channel formation was verified with ultrasound imaging and SEM (Figure 4a). These microchannels in dense native collagen matrices could play an important role in hypoxia/perfusion limitations and the transportation of nutrients, potentially forming blood vessels through dense implants [51]. Later, a study by Esmaeel Sharifi et al. developed sub-micron fiber (with and without Cu content)–collagen/gelatin composite scaffolds. Sub-micron BG fibers were fabricated by the combination of sol–gel and electrospinning processes; the fibers were then mixed with a hydrogel matrix containing gelatin and collagen, freeze-dried, and followed by genipin (incubation in a 0.5% genipin solution) cross-linking to fabricate the final composite scaffolds. A porous structure ranging between 70 and 200 µm was confirmed by the SEM analysis [56]. Cellular biocompatibility tests illustrated that the scaffold containing copper ion in the bioglass structure had more cell growth and osteoblast attachment in comparison to the copper-free scaffolds, indicating non-cytotoxicity and an ideal surface for osteoblast attachment, growth, viability, and bone regeneration.

Even though there are still challenging aspects to overcome before the total commercial exploitation of these composite resorbable materials, the commercialization of similar products has already started from one end. Evonic (Essen, Germany) recently launched composite polymers with precise degradation rates and mechanical properties matching bone-fixation applications [57]. By integrating the osteoconductive properties of calcium phosphate-based additives into its market-leading RESOMER portfolio of bioresorbable polymers, Evonik claims to support medical device customers worldwide to enhance the performance of orthopedic applications that heal or grow bones.

In the following section, we discuss the studies conducted on doping metallic and other therapeutic ions within the phosphate glass composition matrix to understand its impact on fiber mechanical properties and corresponding biomedical applications. Considering these doped fibers to reinforce biopolymers can significantly impact future biomedicine.

### 3.2. Dopants in Phosphate Glass Systems and Ion Releasing

Many phosphate glass systems developed for biomedical applications are based on the ternary system of P_2_O_5_-Na_2_O_5_-CaO that exists in the human body. Therefore, good biocompatibility and low toxicity are expected. By adjusting the mol% of CaO against P_2_O_5_, it was found to be possible to tailor the solubility, ion-release, and thermal characteristics of the glass. Higher concentrations of CaO reduced the solubility, which correlated with the ion-release profile, and this glass showed an increased Tg value [58].

#### 3.2.1. Effect of Doping on Glass Solubility

The dissolution rates of phosphate glass were found to be dependent on the solution pH and temperature, glass composition, fiber diameter, thermal history of the glass, and the surrounding volume of the dissolving medium [59]. The glass composition is a significant factor, and its effect on the dissolution rate has been reported in several studies by Bunker et al., Franks et al., Delahaye et al., and Massera et al. [60,61,62,63]. Many dopants, such as iron, magnesium, copper, boron, and titanium, in the ternary glass system increase the glass’s durability, improving tissue regeneration and cell proliferation [18,64,65,66]. A review that was recently published by Agata Lapa et al. focused on the therapeutic ion release capability of doped phosphate glass fibers [64]. Some information from this review is modified and summarized in Table 4 below. The cationic dopants with higher valence decrease the dissolution rate to a greater extent than cations with a lower valence. Also, cations with small ionic radii have a greater effect on reducing the dissolution rate.

When Fe is doped as Fe_2_O_3_ in the ternary glass matrix, Fe replaces the P in the P-O-P network and creates strong cross-linking P-O-Fe units, whereas the increased MgO and CuO content in the glass replaces the monovalent Na^+^ ions with divalent Mg^2+^ or Cu^2+^, creating more cross-links and making the glass chemically stronger, and thus reducing the leaching rate [75]. The addition of Ti forms the covalent Ti-O-P bonds or ionic cross-links between phosphate chains, increasing the glass characteristic temperatures as well as reducing the solubility rate [76]. Pickup et al. and Foroutan et al. created quarternary Ti-doped (20–30 mol%) phosphate glasses via a sol–gel method, where the same amount of TiO_2_ incorporation was previously not possible with the traditional melt-quenching techniques [77,78]. The dissolution studies conducted in deionized water showed a slower degradation for a higher T_i_O_2_ content, demonstrating the potential for drug-delivery applications.

In the case of Sr, its substitution with Ca forms weaker Sr-O bonds due to the larger ionic radius, reducing the chemical stability of the glass. For these reasons, Sr-doped glass was found to be more soluble than Mg-doped glass [79]. The degradation time of the glasses can be controlled from a few hours to several weeks by adequately tailoring the composition of the glass. In contrast to silicate bioactive glasses, the concurrent release of phosphate-based bioactive glasses is linear with time. This is favorable in biomedicine, where a sustainable, long-lasting release of therapeutic ions into a physiological medium is desired to promote biological functions, such as osteo- and angio-genesis or antibacterial activity [80].

#### 3.2.2. Antibacterial Potential of Doped CaP Glass

Cu- and Ag-doped phosphate glass systems have been widely studied for their antibacterial properties. The idea of releasing the Cu ions from phosphate glasses was investigated a considerable time ago as a control measure for schistosomiasis. They used the controlled release of Cu to create a toxic environment for snails, and the idea was to use the controlled-release glass (CRG) as a molluscicide to kill the mollusks that carried the disease [81]. Later research has demonstrated the potential of Cu-doped calcium phosphate glasses as bioresorbable materials to prevent postsurgical infections, killing opportunistic pathogens and supporting bone tissue regeneration with the controlled release of Cu^2+^ ions. In a study by Mulligan et al., the effect of an increasing amount of Cu^2+^ added to different Na-Ca-P glass compositions was investigated [82]. A clear correlation between the copper content and colony-forming units (CFUs) was observed at least in the first 24–48 h of culture; however, the difference was diminished with further culture time, and was thought to be due to the formation of a biofilm on the glass surface, inhibiting the ion release from the glass.

Mulligan et al. also conducted studies on Ag-doped CaP glass, and the antibacterial effect of the glass was tested [83]. The bacterial viability of *Streptococcus sanguinis* decreased in the first hours after exposure to the glass samples and then started to increase again, as explained in the case of Cu above. This reduction and recovery pattern is due to the silver initially killing the bacteria, eventually forming a layer of them, preventing the release of further Ag ions, and creating a bacterial recovery phase again. However, the glass containing 15 mol% silver gave the highest long-term suppression of bacterial regrowth, proving that the amount of silver released from this composition was sufficient to diffuse and continue killing the bacteria. I Ahmed et al. showed that 3–5 mol% of Ag-doped phosphate glass systems were sufficient to mount a potent antibacterial effect against the test organisms of *S. aureus*, *E. coli*, and *C. albicans* [74]. Overall, the 3 mol% Ag-incorporated 2 mm thick and 5 mm diameter PBG disks investigated in the study were sufficient to mount a potent antibacterial effect against the test organisms, and these compositions also provided an excellent long-term release of the ions into the medium.

In another study by E. A Abou Neel et al., Cu-containing (up to 10 mol%) CaP glass fibers were prepared using a fiber-drawing method and tested against the opportunistic pathogen Staphylococcus Epidermidis. The addition of CuO into the glass fibers was effective in reducing the number of bacteria attached to the fibers [65].

**Figure 5:**
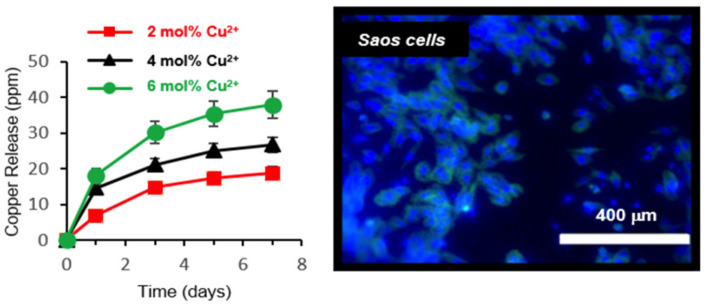
The Cu^2+^-releasing profile from the Cu-doped phosphate glass and the cytocompatibility assessment test on glass particles show a significant increase in the number of cells in the seeded Saos cells in five days. The nuclei are shown in blue color. (Reprinted with permission from [84], copyright 2019).

In a study by Foroutan et al., novel calcium phosphate glasses containing 0, 2, 4, and 6 mol% Cu^2+^ were synthesized via a room-temperature precipitation reaction in an aqueous solution [84]. All samples prepared showed good thermal stability, being amorphous up to ∼530−600 °C depending on the copper content. The antimicrobial effect of Cu^2+^ release was investigated against the *Gram-positive bacteria S. aureus*, and the cytocompatibility assessment was carried out by putting all the synthesized glasses in contact with Saos-2 osteosarcoma cells for a period of 5 days. Osteoblast-like cells *Saos-2* attached and spread on the surface of all glasses over a period of 5 days. However, it has to be noted that a decrease in cell viability was observed with an increasing Cu^2+^ concentration. This could be explained by the fact that high doses of Cu^2+^ increase the reactive oxygen species (ROS), leading to a decrease in cell viability. Thus, a Cu amount of 2 mol% seems to be the optimal composition for bone tissue engineering applications to avoid the complications of cytotoxicity. The antibacterial activity of the glass increases in proportion to the amount of Cu in the glass (Figure 5). The mild and aqueous-based glass-preparation technique used in this study allows for the avoidance of both high-temperature melting and the use of the organic solvent required for the sol–gel technique. In another recently reported study, optically transparent 1 mol% CuO-doped phosphate glass was prepared via melt-quenching and tested for its biocompatibility and antibacterial and viral properties. Despite the low content of Cu in the glass composition, it was found to be effective against bacteria, and the glass surface acted as a trap for SARS-CoV-2, reducing its survival rate [85].

Recently, the ionic doping of bioactive glasses prepared by the sol–gel method has been gaining attention, where the low processing temperature of the sol–gel method allows for a higher loading content of these ions compared with traditional melt-quench techniques.

Sol–gel phosphate-based glasses have been doped with Ga and Sr in different studies [86,87]. The Ga composition resulted in a more consolidated structure with longer phosphate chains, and also provided significant antibacterial activity against *Staphylococcus aureus* compared with the non-Ga-doped glass. More information on doped sol–gel phosphate glasses can be found in [27]. Cerium-doped CaP glass was also tested for its antibacterial potential in a study by Agata Łapa et al. [88].

Lately, Daniela Carta et al. presented a series of cotton-like copper-doped phosphate-based glass fibers in the P_2_O_5_-CaO-Na_2_O-(CuO)_X_ system (x = 0, 1, 3, and 5 mol%) prepared for the first time via the electrospinning of coacervate precursors [89]. The fibers possessed the multifunctionalities of controlled delivery, biocompatibility, and antibacterial properties.

The synthesis of the coacervate occurs at room temperature and in an aqueous solution, allowing for the incorporation of temperature-sensitive molecules. Moreover, electrospinning is an inexpensive, sustainable, and easily scalable manufacturing process. The fibers were fully amorphous, with an average diameter of 1–3 μm. Dissolution tests have shown that the release of phosphate anions ([PO_4_]^3−^, [P_2_O_7_]^4−^, and [P_3_O_10_]^5−^) and cations (Cu^2+^, Na^+^, and Cu2^+^) occurs predominantly within the first 24 h, suggesting that Cu^2+^ acts as cross-linker between phosphate chains. Biocompatibility studies suggested that Cu^2+^ positively affected the viability of MG63 cells, and antibacterial studies showed that Cu^2+^ release effectively killed both Gram-positive and negative bacteria.

#### 3.2.3. Doped CaP Glass in Dental Applications

Fluoride-releasing CaP glass has been previously developed for oral healthcare applications, where releasing fluoride into the oral cavity helped in the remineralization of caries. The slow release of fluoride ions from the implanted dissolving glass pellets provided long-term elevation of salivary F concentrations, preventing dental caries in children [69]. A higher amount of phosphate in the fluoride-releasing glass increased the apatite formation significantly [90]. The toothpaste product BioMinF, launched in 2014 by BioMinF Technologies Ltd. (London, UK), contains bioactive glass patented with a composition of SiO_2_-CaO-Na_2_O-P_2_O_5_-CaF_2_. BioMinF slowly releases calcium, phosphate, and fluoride ions over a timeframe of 8 to 12 h to form a fluorapatite mineral on the dentine for long-lasting protection, which is even more resistant to acid attack than HCA [91].

JC Knowles et al. reported on Sr- and Ca-containing Ti-stabilized phosphate-based glasses prepared via a facile melt-quench technique [92]. Ti was incorporated at a fixed concentration to prolong degradation and Ca/Sr was incorporated to promote osteoinduction. The materials possessed an ideal degradation rate suitable for tissue engineering applications. They showed high cytocompatibility, demonstrating the potential of Sr- and Ti-containing phosphate-based glasses for the treatment of osteoporosis, fractures, and other orthopedic, maxillofacial, and dental applications.

#### 3.2.4. Doped CaP Glass in Veterinary Applications

The research on the intra-rumen delivery of inorganic ions using controlled-release glasses has been ongoing since 1976. Cu and Co are essential micronutrients in animals, and their delivery in cattle and sheep using controlled-release glass has been studied previously [93]. This method was found to be economical and effective for long periods to avoid the necessity for repeated supplementation. The methods such as parenteral treatments and oral supplements are effective for only the short term, and in the former method, the amount which may be administered as a single dose is limited by problems of possible acute systemic toxicity and localized tissue damage. In the CRG systems, the glass with supplements is designed to reside in the animal’s stomach and give a sustained release of elements with a time window from several months to years [94].

The Cu-CRG bolus and Co-CRG bolus were prepared and administered in sheep and cattle [93]. The Cu-containing bolus for cattle weighed approx. 75 g and contained 17.8% copper by weight. It measured 2.5 cm in diameter and 5 cm in length. The sheep bolus weighed approx. 17 g with dimensions of 1.5 cm in diameter and 3.5 cm in length. The cobalt boluses contained 1.8% cobalt by weight and their dimensions were similar to the copper boluses. The animals were slaughtered at varying times and the forestomaches were searched to recover the boluses, which were washed, dried, and weighed. The mean weight of the trace elements released per day was calculated. The minimum rates of the release of cobalt were 0.15 mg of Co/day and 0.85 mg of Co/day in grazing sheep and cattle, respectively. The release continued up to 276 days post-dosing. For the copper boluses, the minimum release rates were 2.2 mg of Cu/day for grazing sheep and 8.0 mg of Cu/day in housed cattle during the following 272 days. The use of Cu- and Co-containing CRG boluses thus appear to be an efficient and practical method of providing long-term supplementation within the recommended daily dietary allowance in sheep and cattle.

However, concerns have been raised due to cobalt-associated toxicity resulting from wear, corrosion, and ion leaching from CoCr implants. Like copper, cobalt also causes oxidative damage to cells by ROS. An increased soluble Co^2+^ ion level might cause serious adverse reactions to the surrounding tissues, as well as systemic toxicity [95]. Co^2+^ ions can activate and increase bone-resorbing osteoclast cell differentiation, resulting in osteolysis aseptic implant loosening. The dose-dependent cytotoxicity of Co has been reported in other studies, where the hydroxyapatite nanoparticles (HANPs) substituted with 5 to 12 wt.% substantially decreased osteoblast cells in vitro. The moderate cytotoxicity of cobalt ions and nanoparticles has been demonstrated previously in various in vitro studies, and Co^2+^ levels of around 400 μM were shown to correspond to IC50 values for apoptosis of C6 glioma cells [96]. Even though the released Co^2+^ ions could be rapidly cleared from the body, unlike in the cell culture conditions, an optimum doping that finds a balance between bioactivity and cytotoxicity should be adapted.

### 3.3. CaP Glass-Based Drug- and Gene-Delivery Systems

In most of the studies mentioned above, the CaP glass or fiber was produced by high-temperature melting followed by drawing. The process of quenching a melt consisting of oxide precursors restricts the types of molecules that can be incorporated into the glass composition, and it limits the use of phosphate-based glass in drug-delivery devices. The use of the low-temperature sol–gel glass preparation methods has circumvented this limit to an extent, and studies were reported on sol–gel phosphate-based glasses for drug-delivery applications [27].

In a study conducted by David M Pickup et al., sol–gel phosphate glass (30CaO-20Na_2_O-50P_2_O_5_) was loaded with the chemotherapy agent cisplatin, a widely used and effective cytotoxic agent in the treatment of malignancies of lung, head and neck, and ovarian cancers, whose efficacy is expected to be significantly enhanced by targeted delivery [97].

For the sol–gel glass preparation, authors used a 1:1 molar mixture of mono- and disubstituted n- butyl phosphate (OP(OH)_2_(OBu^n^) and OP(OH)(OBu^n^)_2_) as the phosphate source and Ca-methoxy-ethoxide (20 wt% in methoxyethanol) as the calcium precursor. The gels were dried at 60 °C and 120 °C for one and two weeks, respectively. X-ray absorption fine structure (EXAFS) spectroscopy was conducted to verify proper drug encapsulation, and UV–visible spectroscopy was conducted to monitor its release into an aqueous NaCl medium. Sustained in vitro release was observed over 4 days. This material has the potential to be prepared as microspheres that can be injected directly into the vicinity of the tumor so that they become trapped in the smaller blood vessels, sustaining the action of cisplatin through controlled release.

Mesoporous phosphate glass (MPG) and non-porous phosphate glass (PG) were synthesized using the sol–gel method by Daniela Carta et al. In [98], Pluronic (P123, Mn = 5800, Aldrich) was employed with the mixture to obtain the porosity. Calcination was performed at 300 °C for 1 h. To assess the intake of drugs for controlled delivery applications of MPG and PG, an antibiotic that inhibits protein growth called tetracycline hydrochloride (TCH) was added to the samples via impregnation. An amount of 5 mg of glass powder was soaked in 5 mL of TCH solution, followed by stirring and centrifugation to separate impregnated glass particles. The TCH release profile was observed using UV–ViS spectroscopy for 24 h.

**Figure 6:**
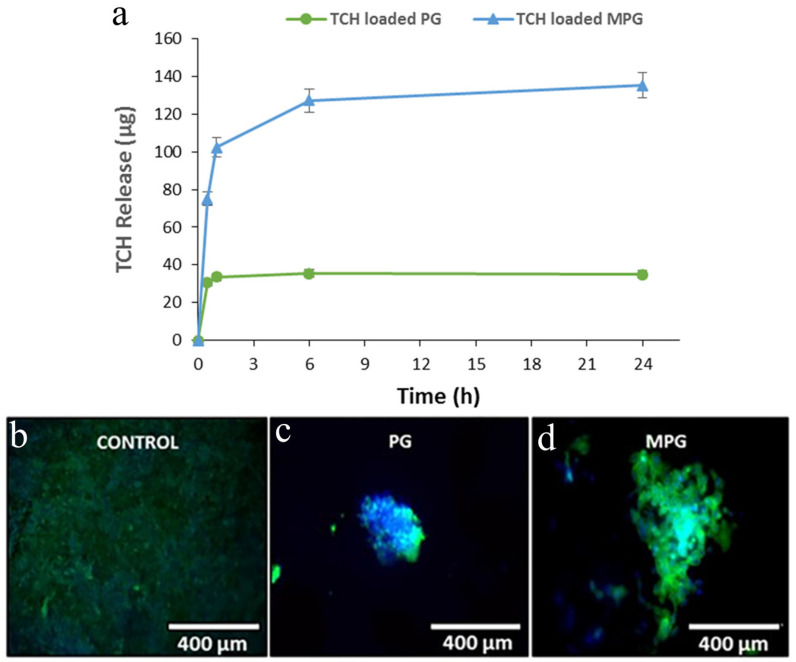
(**a**) Release profiles of TCH as a function of time from loaded MPG and PG samples, where the loaded MPG shows a more sustained release during the 24 h of the study (reprinted with permission from 98). DAPI–Phalloidin staining after 7 days for the (**b**) control, (**c**) non-porous phosphate-based glass (PG), and (**d**) mesoporous phosphate-based glass (MPG) seeded with Saos-2 cells, visualized using a cell image multimode reader. Cell nuclei (blue) and actin filaments (green) show the cells attached and spread on the MPG and PG surfaces after 7 days of seeding (reprinted with permission from 98).

The extended porosity and high surface area of the MPGs allow for a higher loading and more controlled release over time compared to the analogous non-porous system PG (Figure 6). Even though both the MPGs and PGs had similar structures and dissolution rates, the MPGs also showed an enhancement in the HCA formation in the SBF and cell attachment on the glass surface.

Ocean Chaung et al. in [99] reported the fabrication of highly porous amorphous calcium phosphate (ACP) nanoparticles for drug-delivery and bone-healing applications. The synthesis was performed by introducing phosphoric acid to a methanol suspension containing amorphous calcium carbonate nanoparticles at room temperature. These ACP nanoparticles had a short-range atomic order of up to 20 Å and were stable while remaining amorphous for more than one year in air. The ACPs were cytocompatible with bone cells in vitro and showed the release of the alendronate (AL) drug, with a release rate of ~25% of the loaded AL in the first 22 days. Nano-sized CaP particles loaded with growth factors or morphogenetic proteins can enter the cells via endocytosis and dissolve into constituent calcium and phosphate after cellular uptake. This makes them promising competitors against conventional silica, gold, and polymer nanoparticles for gene delivery [100].

Alternatively, Novajra G et al. [72] demonstrated controlled drug release by exploiting the capillary action. This was tested using calcium phosphate glass capillaries fabricated by drawing a preform fabricated by the rotational casting of the molten glass. These capillaries release the solution only from the extremities, unlike the polymer fibers, where the release occurs not only from the fiber ends but also through the fiber walls. The release profile from the hollow fibers can be varied, introducing different types of matrices containing biologically active molecules into the fiber cavity, allowing the tailoring of the release kinetics to match the specific application requirement.

### 3.4. CaP Glass in Soft-Tissue Engineering

Soft tissue regeneration is a vital and promising field where tissues such as nerves, muscles, blood vessels, and fibrous tissues are repaired or regenerated using innovative methods to restore the body’s integrity and normal functionality. CaP glass-based fibers have recently been investigated for soft-tissue engineering applications [101]. Gilchrist et al., 1998, reported the first in vivo evaluation of phosphate glass for a sheep facial nerve repair in 1998. In this study, a phosphate glass tube was developed which was implanted at the epineurium, and it was observed that the glass tube was totally dissolved and the nerves were entirely regenerated in a period of 3 months.

Spinal cord injuries affect the nervous system with severe problems such as neurological deficits and a lack of sensory and motor functions. The primary treatment for those injuries nowadays includes surgery, stabilization of the spinal cord, drug therapy, and rehabilitation. CaP glasses have been studied as a candidate for fixing spinal injuries by Na-Young Joo et al. PGF (50P_2_O_5_-40CaO-5Na_2_O-5Fe_2_O_3_)-containing cylindrical scaffolds of 1.8 mm in diameter and 3 mm in length were developed using the melt-spinning method and implanted into the gap between the proximal and distal stumps following complete transection of the thoracic spinal cord in rats [102]. They found that, from 8 to 12 weeks post-implantation, the locomotor function of the animals and the endogenous brain-derived neurotrophic factor levels in the bladder were improved in rats that received the PGF–collagen scaffolds, which were superior to those of the control group implanted with collagen-only scaffolds. In contrast, axonal growth along the stumps was identified only in the fiber-containing scaffold fibers.

**Figure 7:**
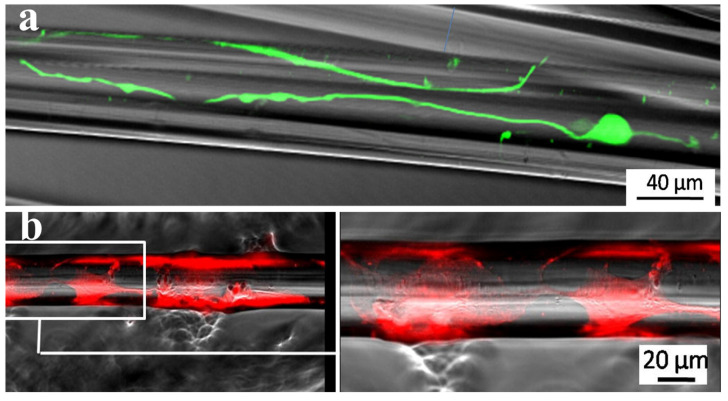
(**a**) DRG neurons on glass fibers extended along the fiber axis direction (reproduced with permission from 104); (**b**) confocal microscope image showing NOBEC (Neonatal Olfactory Bulb Ensheathing cell line) cells spread on the glass surface and enveloping the glass fiber, with the magnified version on the right side (reproduced with permission from 104).

In a study by Young Phil Kim et al. [103], phosphate glass fibers were aligned on compressed collagen rolled into a nerve conduit. In vitro tests on dorsal root ganglion (DRG) neurons and in vivo study on transected sciatic nerves of rats up to 12 weeks showed that the phosphate glass fibers could promote the directional growth of axons acting as physical guides. Similar results were also observed in [104], where both the glial cells and DRG neurons were found to adhere well to the TiPS2.5 glass slices; they grew and extended long axons along the fiber axis direction, providing a directional cue for axonal growth (Figure 7a,b). The tendency for growth along the fiber axis increased with a decreasing fiber diameter.

Many previous studies have also reported that silicate-based bioglass systems with a small percentage of CaO and P_2_O_5_ (1–10%), which have been investigated and found suitable for epithelial and cardiac tissue regeneration [105]. A glass composition called 13-93B3, which contains around 20 mol% of CaO and 2–3 mol% of P_2_O_5_, received approval by the Food and Drug Administration (FDA) and is successfully commercialized for veterinarian medicine as “RediHeal” for skin repair in animals [106].

CaP glasses and fibers have also been used to prepare porous 3D scaffolds for soft-tissue engineering. The fibers with diameters ranging from 10 to 100 microns are randomly placed in a mold during the fabrication so that the free space between them will evolve as the porosity present in the scaffold. A thermal treatment allows these fibers to bond together (sinter) to obtain the glass scaffolds, and the final scaffold structure can be tailored depending on the fiber size, sintering time, and temperature [107]. These scaffolds were loaded with antibacterial ions or growth factors to stimulate repair or soft-tissue regeneration. Bone marrow stromal cells cultured on the scaffolds maintained their metabolic activity and proliferation ability and seemed to be stimulated toward differentiation [108].

Cai et al. proposed the phosphate glass with a composition of 45P_2_O_5_-22CaO-25Na_2_O-8MgO as a reinforcing phase in β-TCP-based scaffolds. These composite scaffolds exhibited enhanced mechanical properties (up to 6 MPa) with respect to pure β-TCP scaffolds (up to 2.3 MPa), as the glass acted as a viscous binder during sintering, thereby strengthening the final scaffold structure to be used in soft-tissue engineering application [109].

Similarly, Ti phosphate glass microspheres in the 10–200 µm size range were produced using a flame-spheroidization apparatus [23]. Irrespective of the TiO_2_ content from 3 mol% to 5 mol%, they provided a surface conducive to the cell growth, attachment, and proliferation of MG63 cells, demonstrating their suitability as substrate materials in bone tissue engineering applications.

## 4. CaP Glass towards the Use in the Fourth Generation of Biomaterials

The field of biomedicine is steadily advancing, and it is now possible to activate or inhibit activities at cellular levels [110,111]. The fourth generation of biomaterials capable of monitoring and manipulating cellular bioelectrical signals is under research [24]. Calcium phosphate glass is currently being studied for development and commercialization as a potential material for advanced biomedical applications [112]. Here, we summarize the recent developments in the optical quality of calcium phosphate glasses and optical fibers, and the proof-of-concept studies on their multifunctional possibilities.

### 4.1. Optical Quality CaP Glass-Based Optical Fibers

The limited depth of penetration of visible light in biological tissues has encouraged research, especially in the past decade, towards the development of optical fibers or waveguides that are made from bioresorbable materials. These implantable optical waveguides make the direct delivery of light possible and support various advanced modalities, such as optogenetics, biological sensing, signal monitoring, and theranostic applications. These implants need not be explanted, reducing the risks and costs associated with the extraction surgeries and reducing biomedical wastes. Biomaterials that have been employed for the fabrication of optical fibers include natural (silk, alginate, cellulose, agarose) and synthetic (hydrogels, PLLA, PLGA, PCL, PDLGA, and inorganic materials such as calcium phosphate glass) materials [113]. The former suffers mainly from limited material sources, poor designability, and low mechanical strength; the latter has rigidity, brittleness, and biocompatibility issues, which are ongoing research areas. The development of an ideal biocompatible material composition with the possibility of tailorable dissolution, good thermo-mechanical properties, and excellent optical quality has been the topic of discussion for the past few decades.

**Figure 8:**
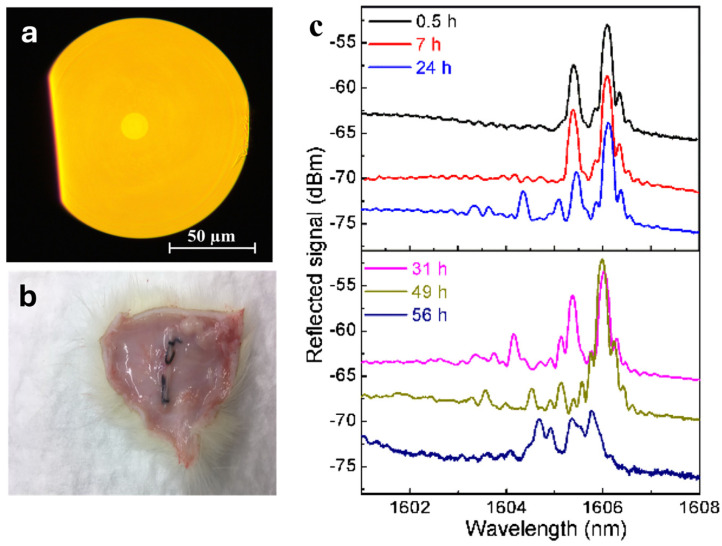
(**a**) Cross-sectional view of the drawn MM bioresorbable fiber.Reprinted with permission from [116] © 2016 Optical Society of America. (**b**) Subcutaneously implanted fiber bundles in rats (reprinted with permission from [117]). (**c**) Reflected signal evolution of a 1° tilted FBG immersed in PBS solution for up to 56 h. The baseline of the different spectra has been adjusted vertically to assist visualization. Reprinted with permission from [118] © 2018 Optical Society of America.

Calcium phosphate glass has been previously explored for its optical properties by altering the glass composition or by adding specific dopant ions [114,115]. A calcium phosphate glass-based optical fiber of high optical quality was reported in 2016 in a study by Ceci-Ginistrelli et al. [116] (Figure 8a). These fibers with a composition of 50 P_2_O_5_ − (30 − x) CaO − (3 + x) MgO − 11.5 Na_2_O − 2.5 B_2_O_3_ − 3 SiO_2_, with x = 0, 5, 12, and 20, were found to be suitable for fiber drawing and were soluble in aqueous media under physiological conditions.

Both the single-mode fiber (SMF) and multimode fiber (MMF) prepared in this study were shown to be effective in guiding light at different wavelengths in the first biological optical window, and the power losses resulted in the lowest measurements in the literature on bioresorbable optical waveguides, as can be seen in Table 5.

From the subcutaneous implantation (Figure 8b) of the 1 cm fiber bundles on adult male rats, these fibers were proved to be resorbable in vivo, demonstrating good biocompatibility with tissues [117]. The skin from the implantation site was histopathologically evaluated on day 14, day 28, and day 35, and no clinical signs of adverse reactions were found in tested animals after the implantation of the bioresorbable fibers. Also, no signs of hepatotoxicity or nephrotoxicity were seen in the liver and kidney parenchyma of the experimental animals after implantation of the bioresorbable fibers. V M Sglavo et al. conducted the prepared fiber’s mechanical characterization, which gave a tensile strength value of around 250 MPa [40]. The presence of MgO content in the composition resulted in the higher stiffness of the glass. The capability of these fibers to work with high-power laser sources was later reported [124]. Both the single and multimode fibers were found to be suitable for high-power laser operation, both in CW and pulsed regimes, paving the way for the employment of these fibers for high-power laser therapeutic applications. The optical fibers withstood without changes in the long-term to the continuous-wave (CW) fiber laser at 1080 nm with an output power up to 13 W, and picosecond laser sources at 515 and 1030 nm with an MW pulse peak power. The picosecond laser sources assessed a laser-induced damage threshold (LIDT) at a fluence higher than 0.17 J/cm^2^.

### 4.2. Optical Quality CaP Multifunctional Devices

Trials have been conducted to accumulate additional functionalities along with the optical quality and biocompatibility of resorbable optical fibers. These include sensors inscribed in the fiber core, channels for drug delivery, and proof-of-concept studies on the suitability of these fibers for diffuse optics-based diagnosis and monitoring of tissue characteristics [118,125,126].

In [118,125], the possibility of writing the fiber Bragg grating (FBG) on phosphate glass fibers was demonstrated. The authors used a 193 nm and 10 ns pulse duration excimer laser. In addition to the inscription of standard FBGs, tilted FBGs with angles of 1° and 0.8° were also successfully recorded. They also observed the change in the reflected signal upon immersion in PBS solution (Figure 8c), and a strength reduction from an initial value of 0.78 dB down to 0.25 dB was assessed after an immersion of 56 h. These fibers were also successfully used for femtosecond laser processing. A 517 nm fs laser was used to direct write plane by plane inscription and grating with a period of 2 micrometers and 1000 periods, giving a grating length of 2 mms. Combining a bioresorbable phosphate glass optical fiber with Bragg grating reflectors paves the way towards developing a soluble class of photonic sensing probes capable of efficiently monitoring vital mechanical or chemical parameters inside the human body.

L. Di Sieno et al. demonstrated the suitability of these bioresorbable fibers for time-domain diffuse optical spectroscopy (DOS) [126]. Optical fibers of a larger core (up to 200 µm) diameter and an NA of 0.17 to maximize the collection efficiency of diffused light were used for this study. They validated the use of bioresorbable fibers on phantoms by applying the MEDPHOT protocol, assessing the capability of measuring the absorption and scattering of homogeneous media. An ex vivo study was conducted by interstitially inserting the bioresorbable fibers into chicken breast tissue to measure the tissue absorption and scattering properties and compare the results with a standard clinical prototype with commercial silica fibers. This study presented a new way of monitoring tissue characteristics inside the body using optical fibers that can be implanted inside the body, which could be helpful in, e.g., tracking the evolution after surgical interventions. The change in light transmission of a 150 µm phosphate fiber of a similar composition during immersion in PBS for 21 days was reported, where the light-delivering capability of the phosphate fiber was not affected by the dissolution for the first two weeks [127]. This demonstrates the potential of phosphate fibers for long-term monitoring applications such as in DOS.

S. H. Mussavi Rizi et al. recently reported a microstructured optical fiber with a core for light delivery and a channel for drug delivery [123]. Extrusion and stack draw techniques were used to fabricate this fiber, demonstrating the potential of this optical-quality phosphate glass composition in the fabrication of complex structures. By changing the drawing conditions, two types of microstructured fibers were produced with a 130 and 230 μm outer diameter and capillary diameter sizes of 17 and 25 μm, respectively. The light guidance was tested, yielding an optical loss of only 0.024 dB/cm, and the microchannel functioning was validated by injecting colored water into the 25 μm channel and observing the flow with an optical microscope. The authors claimed that this type of microfluidic channel is comparable to standard planar channels and allows for a flow of around 10–50 μL/min, which is compatible with the release of drugs or staining agents.

## 5. Summary and Conclusions

We reviewed the advancements in biomedical applications of CaP glass over the past 50 years, observing its persistent role, evolving through all the generations of biomaterials. This glass is biocompatible and bioresorbable, capable of inducing bone or tissue regeneration and axonal growth. Additionally, it can be doped with external therapeutic ions, which are then released in a controllable manner upon the dissolution of the glass. The dissolution rate is tailorable according to the glass composition and can also be used for the controlled release of drugs or genes. We found that these glasses have recently been developed to possess excellent optical transparency, making them attractive among the family of bioresorbable materials for photonic devices. Controllable degradation rates for matched complementary tissue or bone growth are essential to ensure that the material provides support during tissue regeneration. Although glass dissolution rates can be tailored depending on the composition, it does not necessarily mean that a controlled release of the doped ion during the glass dissolution is possible. A better understanding of the mechanism of glass degradation and ion release is needed to ensure that the dosage of ions or drugs released from the glass systems falls within a window of efficacy. Current methods of in vitro tests to understand the dissolution kinetics are over-simplified, as they are not considering the factors related to the dynamic biological environment. The novel techniques of lab-on-a-chip (LOC) could possibly enhance the understanding of the mechanism of degradation by providing a precise control over the fluid flow, mixing, and reaction kinetics within the microfluidic channel of the LOC. To better understand the release profiles of doped ions from the glass matrix, artificial intelligence can also be used where it is possible to analyze data from previous experiments and literature to find the best ratio of dopants, achieving the desired release profile and bioactivity of the glass. Machine-learning algorithms can predict the optimal composition, porosity, and structure of different target tissues. They can also analyze the synergistic effects when multiple ion dopants are added to the glass system, providing insights into how they can improve the biocompatibility or bioactivity.

Along with the understanding of release kinetics, toxicity is another critical challenge to be considered. Sometimes, the entire chemical entity might be toxic to the host tissue, even though the cation itself is not toxic. Current understanding relating to the mechanism of action of metallic ions at the cellular level is still limited and is not well documented. Further investigation with trace metallic cation-doped CaP glasses is going to be very worthy to advance the clinical translation of calcium phosphate glass-based devices. Apart from the biocompatibility assessment studies that are already available in the literature, a comprehensive understanding of the implant by integrating information on their structural characterization and functional assays across multiple time scales could help to cope with the complexity of biological factors. It would also be beneficial to implement real-time monitoring techniques to visualize the implant in vivo, allowing for the early detection of potential issues for timely intervention to ensure stability and functionality.

Innovative methods for synthesizing CaP glass at room temperature are frequently reported, and addressing challenges such as scale-up issues, reproducibility, and cost considerations could facilitate successful commercial production. A comprehensive assessment is needed to understand the toxicological profiles of precursors presently used in these methods to understand their effect on health. Considering the progress made so far in calcium phosphate glass-based optical fibers, we envisage that they hold promise for becoming enabling tools for various applications in biomedicine. Beyond the reported proof-of-concept studies on label-free optical sensing and in vivo monitoring, there is indeed an immense potential for exploring bioresorbable fibers for the light-induced manipulation of cells using optogenetics and light-triggered drug release. Combined with modern nanotechnology, their potential in advanced biosensing, such as the optical detection of biomolecules or viruses, could be explored analogously to the studies conducted with commercial silica fibers. Integrating light delivery, drug delivery, and label-free optical sensing and electrical recording into a single bioresorbable fiber can revolutionize the monitoring of the brain and other physiological functions at the cellular level. This multifunctional fiber could play a crucial role in the diagnosis and therapy of tumors, as well as in monitoring the healing process and providing information over an extended time. There is also a need to develop efficient connectors for fiber termination to facilitate the clinical translation or commercialization of CaP glass-based optical fibers. These connectors would enable the connection or disconnection of the bioresorbable fiber from a commercial silica fiber that remains outside the patient’s body. The evidence is pretty convincing that the CaP glass-based systems, which were just a supporting material for bone growth for decades, have advanced to the point where they can now perform several biomedical functions. We hope the research on this topic will intensify in the coming years to meet the challenges of bringing them from research to the market.

## Figures and Tables

**Table 1:** Comparison of mechanical properties of bone tissue with widely used metallic polymer and phosphate glass implants.

	Flexural Strength (MPa)	Elastic Modulus (GPa)	Reference
Cortical bone tissue	70–150	7–30	[6]
Ti and Ti alloys (metals)	960–1100	105–125	[37]
PLA (polymer)	90	3.8	[39]
Phosphate glass fiber	366	51	[40]

**Table 2:** Main studies on the long-term stability and biocompatibility of CaP glass–ceramic implants during 15 years, followed by the CaP formulation of L.L. Hench in 1970.

Author And Year of Study	Glass Composition	In Vitro Tests	In Vivo Tests	Main Results
L.L. Hench, 1971 Ref: [13]	- (45-55)-SiO_2_-P_2_O_5_-CaO-Na_2_O. - (Ca/P molar ratios of 5.0 and 1.8). - Prepared by high-temperature melting (1450 °C).	- At a PH = 5.4 (buffered with succinic acid and NaOH). - Temp = 37 °C. - Duration of up to 9 months.	- Millimeter (mm) size of ceramics with a Ca/P molar ratio of 5. - Implanted in rat femurs. - Duration of 6 weeks.	- A self-protecting in vitro solubility behavior with an equilibrium PH of 10 observed for up to 9 months. - Intimate bonding between glass ceramic and bone. No histologic evidence of inflammatory response.
B.A. Blencke, 1976 Ref: [28]	- Stabilized ceramic, ceravital, and high-alkaline glasses. - Ceravital/SiO_2_(40-50)-P_2_O_5_(10-15)-CaO(30-35)-Na_2_O(5-10)-K_2_O-MgO. - Stabilization by addition of Al_2_O_3_ and TiO_2_. - Prepared by high-temperature melting (1500 °C).	- At a PH = 7.3 in water. - On powder samples of all three types of compositions.	- Glass ceramic implants in mm size. - Implanted in tibia or femur of the rats, rabbits, and German Shepherds. - Period of up to 30 months.	- High-alkaline glass showed highest solubility both in vitro and in vivo. - Glassy implant was destroyed by the body fluids over a duration of 20 months. - Intimate junctions between the glass–ceramic implant and the bone were evident in the implant period. - No evidence of inflammation or osteoclastic reactions.
Hisao Fukui, 1977 Ref: [29]	- CaO (55 mol%) and P_2_O_5_ (45 mol%). - Prepared by high-temperature melting at 700 °C.	- -	- Ceramic specimen implant in right femurs of 15 male rats, mm size. - Duration of 6 weeks. - Titanium wire controls implanted on the left femur.	- By 6 weeks, histologically, the titanium implant space was surrounded by fibrous connective tissue. - The ceramic implant space faced the mature lamellar bone. - The Ti control implant was still movable, but the ceramic implant was better fixed at the position.
J. Burnie, T. Glichrist, 1981 Ref: [31]	- P_2_O_5_ (35–60 mol%) as the network former. - Prepared by high-temperature melting (1100 °C).	- Cell culture studies against L929 mouse fibroblast cell line. - 7-day minimum test period.	- Subcutaneous and intramuscular implantation of CRG disks and rods. - Implanted in the tibia of adult sheep up to 3 months.	- No cytotoxic effects of CRG in tissue culture and a very limited reaction at soft-tissue implant sites. - CRG dissolved in vivo, allowing normal bone healing and repair.
Graves, Jr., 1986 Ref: [30]	- 23CaO-77 P_2_O_5_. - High-temperature melting (900 °C).	- Fibers tested in buffered saline static solution. - Duration of 40 days.	- PLA-coated fiber (around 70 µm) implanted in rats and rabbits for a maximum duration of 24 weeks.	- Tensile strength of fiber: 255 MPa. - Complete in vitro dissolution in 40 days. - Fibers slowly degraded and consumed in the physiological environment with evidence of inflammation.

**Table 3:** Mechanical properties and biomedical applications of CaP fiber-reinforced composites.

Composite	Fiber Composition	Mechanical Property	Application	Ref
PLA-PBG fiber composites	50 P_2_O_5_-40CaO-5Na_2_O-5Fe_2_O_3_	Y: 5 GPa F.S: 90 MPa	Bone fracture-fixation devices	[49]
Methacrylate- modified oligolactide-PBG fiber	35 P_2_O_5_–27.5 CaO–9.5 MgO–22.5 Na_2_O–5.5 TiO_2_	Y: 16 GPa F.S:115 MPa	Bone-fixation devices	[48]
PLA-PGF (UD)	40P_2_O_5_−24MgO−16CaO−15Na_2_O−4Fe_2_O_3_	Maximum flexural load PLA: 200 N PLA-PGF: 400 N	Bioresorbable screws	[45]
PLA and PBG	50P_2_O_5_–40CaO–5Na_2_O–5Fe_2_O_3_	Y: 18 Gpa F.S: 190 Mpa	Intramedullary rods	[39]
	40P_2_O_5_–24MgO–16CaO–16Na_2_O–4Fe_2_O	Y: 26 Gpa F.S: 240 Mpa		
Polyester–PGF	52P_2_O_5_-24CaO-5K_2_O-13MgO-1TiO_2_-5Fe_2_O_3_	Y: 3 GPa F.S: 43.8 MPa	Orthopedic implants	[50]

PBG: phosphate-based glass; Y: elastic modulus; F.S: flexural strength, PGF: phosphate glass fiber.

**Table 4:** Dopants to the ternary phosphate glass system, their effect on the glass, and the respective biomedical applications.

**Dopant**	**Effect on Phosphate Glass**	**Fiber Composition**	**Biomedical Application**	**Ref**
Iron (Substitute of Na_2_O)	Increased Tg and chemical durability, and decreased dissolution rate	P_2_O_5_ Na_2_O CaO Fe_2_O_3_	Better cell adherence and proliferation	[18]
Copper (Substitute of Na_2_O)	Increased Tg and durability, and decreased dissolution rate	P_2_O_5_ Na_2_O CaO CuO	Antibacterial and wound healing	[66]
Magnesium (Substitute of Na_2_O)	Increased Tg and chemical durability, and decreased dissolution rate	P_2_O_5_ CaO Na_2_O MgO	Polymer reinforcement and bone repair	[67]
Strontium (Substitute of CaO)	Slight decrease in Tg and a significant decrease in Tm	P_2_O_5_ CaO Na_2_O MgO SrO	Osteoporosis	[65]
Boron (Substitute of Na_2_O)	Increased Tg, Tm, and Tc, and decreased dissolution rate	P_2_O_5_ B_2_O_3_ CaO MgO Na_2_O	Bone fixation and growth	[68]
Fluoride (Substitute of CaO)	Lowered Tg and Tx, and slight decrease in mechanical properties	P_2_O_5_ CaO Na_2_O CaF_2_	Oral healthcare	[69,70]
Zinc (Substitute of CaO)	Increased mechanical and reduced degradation properties	P_2_O_5_ Na_2_OCaO ZnO	Treats catheter-associated urinary tract infections	[71]
Titanium (Substitute of Na_2_O)	Increased Tg and Tx and glass stability	P_2_O_5_ CaO Na_2_O TiO_2_	Bone-binding application (increased hydroxy apatite formation)	[22]
Potassium	Higher viscosity and glass durability	P_2_O_5_ CaO Na_2_O SiO_2_ K_2_O TiO_2_	Fibers for nerve tissue regeneration and controlled-release systems	[72]
Aluminum	Increased durability	P_2_O_5_ Al_2_O_3_ ZnO	Muscle engineering (craniofacial)	[73]
Silver (Substitute of Na_2_O)	Decreased degradation	P_2_O_5_ CaO Na_2_O Ag	Antibacterial property	[74]

Tg: glass transition temperature; Tm: melting temperature; Tx: crystallization temperature.

**Table 5:** Optical losses reported in various optical fibers fabricated from different biomaterials.

Material	Fabrication Technique	Fiber Type	Optical Loss	Reference
Spider silk	Native spider silk directly woven by spiders	Unclad fiber	10 dB/cm at VIS	
Cellulose	Core was produced using dry-jet wet spinning in a water bath as a coagulant. Cladding produced by coating the core with cellulose acetate dissolved in acetone	Core–cladding fiber	10 dB/cm at 750–1350 nm	[113]
Agarose	Boiled agar solution poured into the glass mold tube with rods, cooled down, and released after solidification	Structured fiber Core: 0.64 mm Clad: 2.5 mm Airholes: 0.5 mm	3.23 dB/cm at 633 nm	
PLLA	Thermal drawing process of PLLA crystalline powders melts at 220 °C	Unclad fiber	1.6 dB/cm at 473 nm 4.8 dB/cm after 40 days of soaking in water	[119]
PCL	Thermal drawing of the preform in the drawing tower	Unclad solid-core and grooved fibers	1.5 dB/cm at 635 nm in PBS	
PCL	Fiber draw tower	PCL capillary	2 dB/cm at 635 nm over 21 days immersion in PBS	[120]
Wrapped spider silk on tapered SMF-28	Heating of SMF and followed by wrapping the silk over the biconical fiber shape	Tapered commercial silica fiber with silk wrapping	−32 dB at 1360–1390 nm (measured by Optical Spectrum Analyzer)	[121]
Phosphate glass fiber	Rod in tube technique	Core–cladding MMF Core–cladding SMF	0.019 dB/cm at 1300 nm 0.047 db/cm at 633 nm	[116]
Phosphate glass fiber	Stacking of the extruded tube within the extruded capillary, followed by thermal drawing	Microstructured	-	[122]
Phosphate glass fiber	Stack and draw	Core–clad for light delivery and channel for drug delivery	0.024 dB/cm at 1300 nm	[123]

## Data Availability

No new data were created or analyzed in this study. Data sharing is not applicable to this article.
